# Gut microbiota, circulating metabolites, and pancreatic cancer risk: a multi-method causal inference study with cross-population validation

**DOI:** 10.3389/fmicb.2025.1730313

**Published:** 2026-01-23

**Authors:** Shicheng Lin, Enze Shi, Yuxin Zhang, Xiaofan Wang, Zhen Tian, Jing Han, Quanwang Li

**Affiliations:** 1 Graduate School, Beijing University of Chinese Medicine, Beijing, China; 2 Department of Oncology, Dongfang Hospital, Beijing University of Chinese Medicine, Beijing, China; 3 Department of Laboratory Medicine, Dongfang Hospital, Beijing University of Chinese Medicine, Beijing, China

**Keywords:** 16S rDNA sequencing, gut microbiota, mendelian randomization, metabolites, pancreatic cancer

## Abstract

Pancreatic cancer (PC) is a lethal malignancy with limited early detection strategies and poor therapeutic response. Emerging evidence implicates the gut microbiota in carcinogenesis, yet whether microbial alterations are causal or secondary remains uncertain. In this study, we integrated cross-sectional 16S rDNA sequencing, two-sample Mendelian randomization (MR), and mediation analysis to investigate the causal role of gut microbiota in PC risk. We profiled fecal microbiota in a Beijing-based cohort of 26 newly diagnosed PC patients and 9 healthy controls, revealing significant dysbiosis characterized by reduced microbial diversity, depletion of butyrate-producing genera (e.g., Faecalibacterium), and enrichment of pro-inflammatory taxa such as Olsenella. Using European GWAS summary data, MR analysis identified 17 gut microbial taxa causally associated with PC risk, including Olsenella and Pauljensenia sp000411415. Notably, higher abundance of Pauljensenia sp000411415 was associated with increased PC risk, an effect partially mediated by reduced circulating levels of octanoylcarnitine (C8) and glutarylcarnitine (C5-DC)—metabolites independently linked to lower PC risk. Population-matched MR in East Asian cohorts validated several causal associations, enhancing ancestral relevance. Our findings support a causal role for specific gut microbes in pancreatic carcinogenesis and highlight a Pauljensenia–acylcarnitine axis whereby microbial suppression of protective metabolites may contribute to disease development. This integrative approach bridges microbial dysbiosis with functional mechanisms, offering novel insights for microbiome-informed strategies in PC prevention and early detection.

## Introduction

Pancreatic cancer (PC) is one of the most lethal malignancies worldwide, with a mortality rate nearly equal to its incidence. It ranks as the sixth leading cause of cancer-related death globally (Bray et al., 2024). Due to its insidious onset, the majority of patients are diagnosed at an advanced, surgically unresectable stage. Furthermore, PC exhibits limited responsiveness to conventional therapies—including chemotherapy, radiotherapy, targeted therapy, and immunotherapy—resulting in a dismal prognosis and a 5-year survival rate of only 10% (Wu et al., 2022; Wang et al., 2023). Early detection offers the best opportunity for curative intervention and improved survival, highlighting the urgent need to identify modifiable risk factors that could inform prevention, early diagnosis, and therapeutic strategies.

Accumulating evidence has revealed the presence of a low-biomass intratumoral microbiome in pancreatic tissue—previously considered sterile—implicating microbial communities in tumor progression, diagnosis, and treatment response (Luo et al., 2025). The gut, the largest reservoir of commensal microorganisms in the human body, is believed to be a primary source of these intratumoral microbes, potentially seeding the pancreas via the portal circulation or enterohepatic pathways (Guan et al., 2023; Thomas et al., 2018). Although tumor colonization may originate in the upper gastrointestinal tract, microbial signals from proximal sites can propagate downstream and are detectable in stool (Vilà-Quintana et al., 2024; Tavanaeian et al., 2025; Chung et al., 2021). For instance, *Gemella morbillorum* and *Fusobacterium nucleatum* subsp. *vincentii*—taxa linked to pancreatic tumors (Sidiropoulos et al., 2024)—have also been reported in fecal samples from patients with pancreatic cancer (Chung et al., 2021). While fecal profiles may not fully recapitulate the spatial origin of tumor-associated microbes, their non-invasive accessibility supports their use as a practical surrogate for risk stratification and early detection.

The gut microbiota interacts dynamically with the host, modulating immune responses, regulating inflammation, and influencing carcinogenesis through the production of bioactive metabolites and modulation of signaling pathways (Tilg et al., 2020; Koh et al., 2016). However, microbial composition is highly susceptible to environmental influences such as diet, antibiotic use, chronic inflammation, and nutritional status, raising uncertainty about whether observed microbial alterations in PC are causal drivers or secondary consequences of disease. Moreover, bidirectional crosstalk between the gut microbiome and host metabolism may contribute to tumor metabolic reprogramming. Gut microbes participate in diverse metabolic processes, influence cancer-related signaling cascades, and shape host immune function, thereby exerting either pro- or anti-tumorigenic effects (Meng et al., 2023). Yet, it remains unclear whether circulating metabolites might mediate the influence of gut microbiota on PC susceptibility.

Despite growing interest in the gut–pancreas axis, critical questions regarding causality and mechanism remain unresolved. Large-scale human studies are often limited by confounding and reverse causation, particularly in observational designs. Recently, Mendelian randomization (MR) has emerged as a powerful tool for causal inference (Smith, 2010; Emdin et al., 2017), leveraging germline genetic variants as instrumental variables to mimic randomized controlled trials (Burgess et al., 2023). By minimizing confounding and avoiding reverse causality, MR enables robust assessment of whether gut microbiota alterations are causally linked to PC risk—not merely associated.

However, current research on the causal relationship between gut microbiota and pancreatic cancer remains limited, largely confined to single-cohort studies or observational analyses. Critical gaps include the lack of multi-population validation and systematic exploration of underlying biological mechanisms. For instance, no study to date has systematically integrated microbiome profiling with serum metabolomics to dissect causal pathways along the “gut–pancreas axis” (Chen Z. et al., 2024; Li et al., 2023; Li and Liang, 2024). Moreover, existing Mendelian randomization (MR) evidence is predominantly derived from European populations, with a striking paucity of data from Chinese or East Asian cohorts (Jiang et al., 2023; Zhu and Ding, 2024). This geographical bias severely limits the generalizability of findings to non-European ancestries.

To address these limitations, we integrated 16S rDNA sequencing data from a Beijing-based cohort with international genomic resources, employing two-sample Mendelian randomization (MR) and mediation analysis to assess causal links between gut microbiota and PC. We identified causally associated taxa in European-ancestry populations—such as the pro-inflammatory genus Olsenella and the risk-associated Pauljensenia sp000411415—with effects partially mediated by serum acylcarnitines, whose depletion may contribute to pancreatic carcinogenesis. In East Asian MR analyses, while the specific set of significantly associated taxa differed from that in Europeans, several microbial signals showed directionally consistent effects, suggesting potential shared biological pathways alongside population-specific associations. These cross-ancestry comparisons help contextualize our findings within diverse genetic and environmental backgrounds and highlight the need for inclusive, multi-ethnic studies in microbiome–disease research. This work provides mechanistic insights into the “gut–pancreas axis” and underscores microbial–metabolite pathways as promising targets for early PC detection.

## Methods

### Study approval, registration, and informed consent

**Figure 1:**
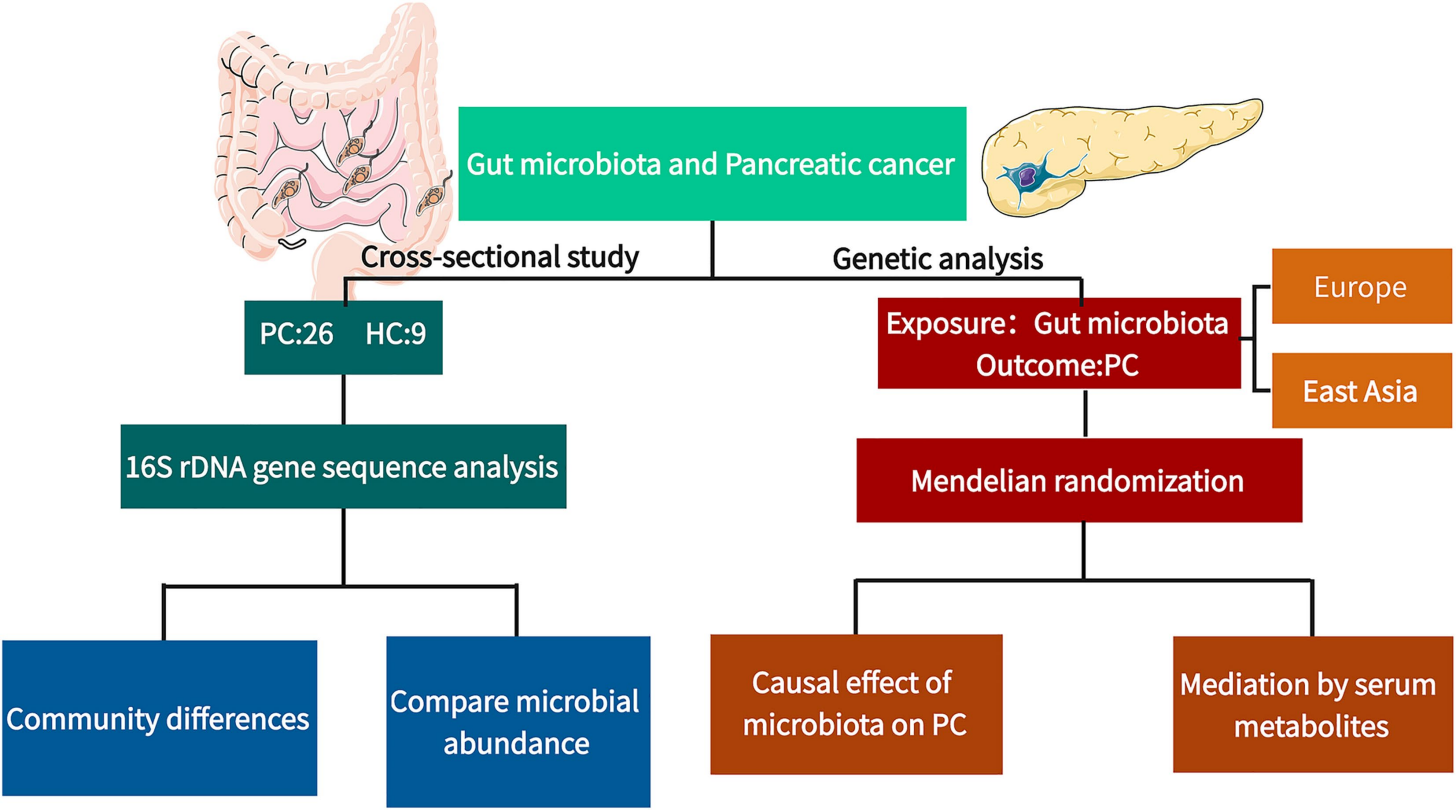
Study design flowchart. This study integrates a cross-sectional 16S rDNA sequencing analysis with two-sample MR to investigate the association between gut microbiota and pancreatic cancer, and to explore causal mechanisms and mediating roles of serum metabolites. PC, pancreatic cancer; HC, healthy controls.

An overview of the study design is presented in Figure 1. This study was approved by the IRB of Dongfang Hospital Beijing University of Chinese Medicine (approval number: JDF-IRB-2024055002) and registered at the Chinese Clinical Trial Registry (ChiCTR, ID: ITMCTR2025001710). Written informed consent was obtained from all participants in accordance with the Declaration of Helsinki.

### Study design and sample collection

We recruited patients with newly diagnosed PC from Dongfang Hospital, Beijing University of Chinese Medicine and Cancer Hospital of the Chinese Academy of Medical Sciences, between October 2024 and May 2025. Healthy controls (HC) were enrolled from community residents and health check-up centers in southern Beijing, matched to PC patients by age, sex, and body mass index (BMI).

Inclusion criteria for PC patients were: (1) histologically confirmed diagnosis of pancreatic cancer; (2) no prior chemotherapy, radiotherapy, targeted therapy, immunotherapy, or other anti-tumor treatments; (3) age 18–75 years; and (4) provision of written informed consent. Controls were free of chronic diseases and gastrointestinal disorders. Exclusion criteria for both groups included: (1) history of malignancy treated with anti-cancer therapies; (2) prior gastrointestinal conditions (e.g., inflammatory bowel disease, peptic ulcer, gastroenteritis, irritable bowel syndrome, or gastrointestinal tumors); (3) use of antibiotics or corticosteroids within the past 2 months; or (4) intake of probiotics or prebiotics within the past week. Demographic data—including age, sex, and BMI—were collected during recruitment. Pancreatic cancer diagnoses were independently verified by two pathologists at Beijing University of Chinese Medicine.

### DNA extraction and 16S rDNA gene sequencing

Total genomic DNA was extracted from fecal samples using the Magnetic Soil and Stool DNA Kit (BioTeke, DP712) following the manufacturer’s protocol. DNA concentration was measured using Qubit (Invitrogen, United States). The V3–V4 hypervariable region of the 16S rDNA gene was amplified via PCR using primers 341F (5′-CCTACGGGNGGCWGCAG-3′) and 805R (5′-GACTACHVGGGTATCTAATCC-3′), under the following cycling conditions: initial denaturation at 98 °C for 30 s; 32 cycles of 98 °C for 10 s, 54 °C for 30 s, and 72 °C for 45 s; followed by final extension at 72 °C for 10 min. PCR products were purified using AMPure XT Beads (Beckman Coulter Genomics), quantified by Qubit, and assessed on an Agilent 2100 Bioanalyzer. Libraries were pooled and sequenced on an Illumina NovaSeq 6000 platform (PE250 mode) at LC-Bio Technology (Hangzhou, China).

### 16S rDNA gene sequence analysis

Raw sequencing reads were demultiplexed and processed using cutadapt (v1.9) and FLASH (v1.2.8) for primer trimming and paired-end read merging. Low-quality sequences (Phred score <20, length <100 bp, or containing >5% N bases) were filtered using fqtrim (v0.94). Chimeric sequences were removed with VSEARCH (v2.3.4). Amplicon sequence variants (ASVs) were inferred using DADA2 for high-resolution taxonomic profiling. Taxonomic annotation was performed using the QIIME2 plugin *feature-classifier* against the SILVA and NT-16S databases (16S rDNA gene, 99% identity). Alpha and beta diversity metrics were calculated in QIIME2. Differential abundance analysis at the genus level was performed using ANCOM-BC2 to account for the compositional nature of microbiome data and control for false discovery rate (FDR). Taxa with an FDR-corrected *q*-value <0.1 were considered statistically significant. All visualizations were generated in R (v3.4.4).

### Gut microbiota and pancreatic cancer GWAS summary data

Summary-level genetic data for gut microbiota were obtained from a genome-wide association study (GWAS) of the Finnish FINRISK 2002 cohort (Qin et al., 2022), a population-based study comprising 5,959 individuals. Genome-wide association testing of 7,979,834 human genetic variants was conducted to identify host genetic determinants of gut microbial composition, as profiled by metagenomic sequencing. This analysis revealed 567 independent SNP–taxon associations, implicating host genetics in the regulation of gut microbiome structure. The study covered six geographic regions in Finland and reported results for 2,801 microbial taxa, classified within 473 GTDB taxa (including 59 phyla, 95 classes, 187 orders, 415 families, 922 genera, and 1,123 species). Comprehensive summary statistics are publicly available in the NHGRI-EBI GWAS Catalog (accession numbers GCST90032172–GCST90032644). The original study was approved by the Coordinating Ethics Committee of the Helsinki and Uusimaa Hospital District (reference number 558/E3/2001). Pancreatic cancer data were obtained from FinnGen release R12 (pancreatic malignant neoplasms, MNP; cases = 3,139, controls = 378,749).

Gut microbiome and metabolic pathway data for Asian populations were obtained from the China Nucleotide Sequence Archive (CNSA) (Liu et al., 2022). The pancreatic cancer outcome dataset for East Asian populations was sourced from Biobank Japan, with identifier GCST90018673 (Sakaue et al., 2021).

### Mendelian randomization analysis

We employed the TwoSampleMR R package^1^ to assess causal relationships between gut microbiota and pancreatic cancer risk. Forward MR analyses were conducted with microbial taxa as exposures and PC as the outcome; reverse MR analyses tested the inverse direction. Instrumental variables (IVs) were defined as genome-wide significant (*p* < 1 × 10^−5^) SNPs with minor allele frequency (MAF) >0.01. To minimize weak instrument bias, only SNPs with *F*-statistics >10 were retained. Clumping was performed to ensure independence (*r*^2^ < 0.001, window size = 10 Mb).

The inverse-variance weighted (IVW) method was used as the primary approach to estimate causal effects. Continuous outcomes are reported as beta coefficients (*β*) with standard errors; binary outcomes are presented as odds ratios (ORs) with 95% confidence intervals (CIs). Significance was set at *p* < 0.05. Heterogeneity was evaluated using Cochran’s *Q* statistic (*p* < 0.05 in IVW or MR-Egger indicating heterogeneity). Horizontal pleiotropy was assessed via the MR-Egger intercept test; a significant deviation from zero (*p* < 0.05) indicated presence of pleiotropy. Effect estimates and 95% CIs were derived using fixed-effect IVW models.

### Mediation analysis of gut microbiota, serum metabolites, and pancreatic cancer

To investigate whether circulating metabolites mediate the gut microbiota–PC association, we performed a two-step Mendelian randomization mediation analysis. Genetic instruments for serum metabolites were obtained from a large-scale GWAS of blood metabolomics in European populations (Sollis et al., 2023) (GWAS Catalog: GCST90199621–GCST90201020), encompassing 1,091 individual metabolites and 309 metabolite ratios after stringent quality control (Chen et al., 2023).

The total effect (*β*_EO_) of gut microbiota on PC risk was decomposed into: (1) the indirect effect mediated through a given serum metabolite, estimated as *β*_EM_ × *β*_MO_, where *β*_EM_ is the causal effect of the microbial exposure on the metabolite, and *β*_MO_ is the causal effect of the metabolite on PC; and (2) the direct effect. The proportion mediated was calculated as (*β*_EM_ × *β*_MO_)/*β*_EO_. All mediation tests were corrected for multiple testing using the false discovery rate (FDR) method.

## Results

### Demographic and clinical characteristics

A total of 26 patients with PC and 9 HC were included in this study. All participants were of Han Chinese ethnicity and resided in northern China, where the typical diet includes wheat, rice, meat, vegetables, and legumes. Although dietary patterns varied among individuals, none of the participants were vegetarians. Demographic and clinical characteristics are summarized in Table 1. No significant differences were observed between the two groups in terms of sex, age, BMI, smoking status, alcohol consumption, or history of diabetes (*p* > 0.05).

**Table 1:** Demographic and clinical data.

Characteristic	PC (*n* = 26)	HC (*n* = 9)	*p*
Gender (female/male)	12/14	5/4	0.711^a^
Ages (years)	65.69 ± 8.24	59.78 ± 6.61	0.061^b^
BMI	22.29 ± 3.00	23.07 ± 2.15	0.482^b^
Smoking (yes/no)	8/18	1/8	0.391^a^
Drinking (yes/no)	5/21	2/7	1.000^a^
Diabetes (yes/no)	8/26	1/8	0.657^a^

BMI, body mass index.

a Kruskal–Wallis test.

b Chi-square test.

### Gut microbiota dysbiosis in pancreatic cancer patients

**Figure 2:**
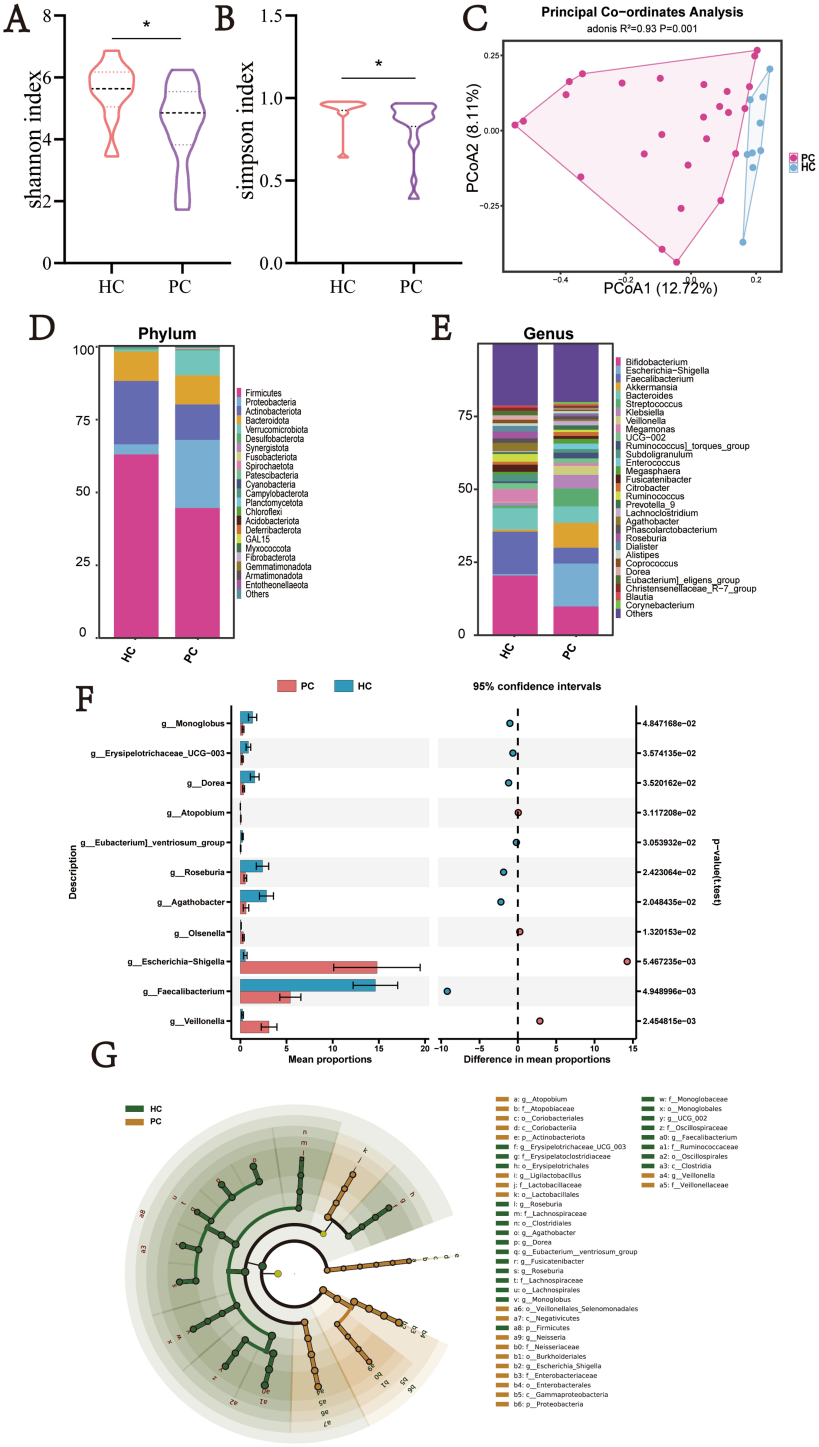
Bioinformatics analysis of 16S rDNA gene sequencing data from a cross-sectional cohort of pancreatic cancer patients (PC, *n* = 26) and healthy controls (HC, *n* = 9). **(A)** Shannon index and **(B)** Simpson index were significantly lower in the PC group (*p* < 0.05), indicating reduced alpha diversity at the ASV level. **(C)** PCoA based on Bray–Curtis dissimilarity revealed significant separation of microbial community structure between groups (PERMANOVA, *R*^2^ = 0.07, *p* = 0.001), indicating altered beta diversity in pancreatic cancer. **(D)** Relative abundance of gut microbiota at the phylum level. **(E)** Relative abundance of gut microbiota at the genus level. Taxonomic composition was visualized using stacked bar plots, showing distinct microbial profiles between PC and HC groups. **(F)** Differential abundance plot showing mean relative abundances and between-group differences of genera identified as differentially abundant by ANCOM-BC2 (*q* < 0.05) in PC patients versus HC. The PC group exhibited significantly lower mean abundance of *g__Faecalibacterium*, whereas *g__Olsenella*, *g__Escherichia–Shigella*, and *g__Veillonella* were markedly enriched. **(G)** Cladograms depicting the phylogenetic relationships among taxa identified as differentially abundant by ANCOM-BC2, with branches colored according to the group (PC or HC) in which each taxon is enriched.

To investigate gut microbiota differences between PC patients and HC, we collected fecal samples from individuals at Dongfang Hospital, Beijing University of Chinese Medicine, and the Cancer Hospital of the Chinese Academy of Medical Sciences. 16S rDNA sequencing was performed to profile microbial composition. Alpha diversity analysis revealed that, compared with the HC group, the PC group exhibited significantly lower Shannon and Simpson indices (*p* = 0.028 and *p* = 0.018, respectively; Figures 2A,B), indicating reduced microbial diversity, while no significant differences in community richness were observed between the two groups (Supplementary Figures S1A–C). Principal coordinates analysis (PCoA) was used to assess overall microbial community structure, and analysis of similarities (ANOSIM) revealed a significant separation between the PC and HC groups (*p* = 0.001, Figure 2C), suggesting marked gut microbiota dysbiosis in PC patients.

Further taxonomic analysis revealed significant alterations in gut microbial composition between PC patients and HC. At the phylum level, the PC group exhibited decreased relative abundances of Firmicutes, Actinobacteriota, and Bacteroidota, accompanied by a notable increase in Proteobacteria (Figure 2D). At the genus level, PC patients showed a marked depletion of multiple butyrate-producing taxa, including Faecalibacterium, Roseburia, Lachnospira, Fusicatenibacter, Butyricicoccus, and Ruminococcus, while genera such as Veillonella, Megasphaera, Fusobacterium, and Olsenella were significantly enriched (Figure 2E), indicating a shift toward a pro-inflammatory microbial profile and intestinal dysbiosis. To rigorously identify differentially abundant taxa while accounting for the compositional nature of microbiome data, we performed ANCOM-BC2 analysis (*q* < 0.05) (Figures 2F,G). This analysis revealed distinct microbial signatures: in the PC group, Escherichia-Shigella and Olsenella were significantly enriched, taxa often associated with mucosal inflammation and pathogenic potential. In contrast, Faecalibacterium—a well-characterized anti-inflammatory commensal known for its role in maintaining gut barrier integrity and immune homeostasis—was significantly depleted in PC patients. These findings collectively indicate a functionally distinct gut microbiota composition in PC patients compared to healthy individuals.

### Causal relationships between gut microbiota and pancreatic cancer

**Figure 3:**
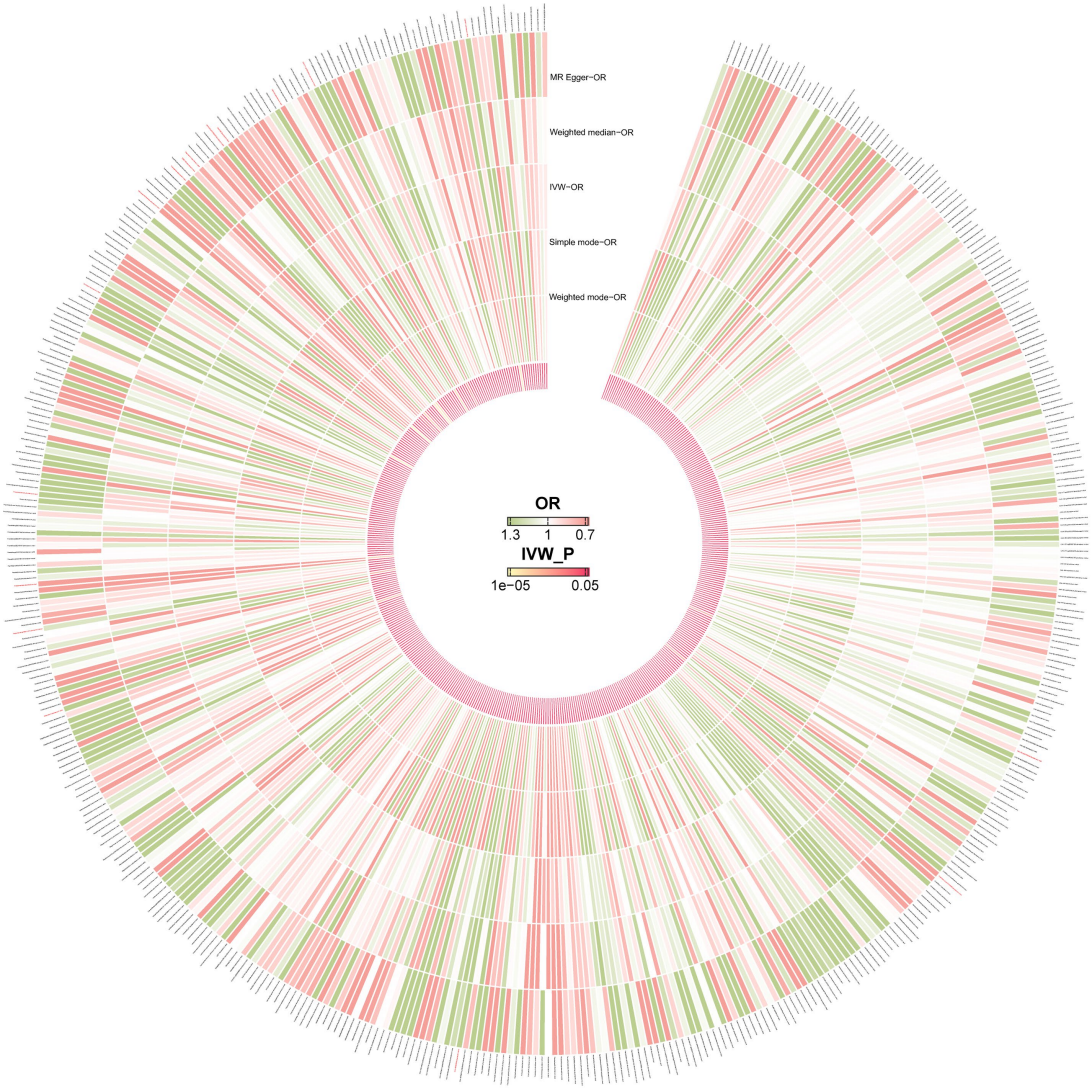
Circular heatmap summarizing the causal associations between gut microbial taxa and pancreatic cancer risk from Mendelian randomization analysis. Each radial segment represents a microbial taxon at genus or higher level, ordered by phylogenetic relatedness. The inner ring displays the odds ratio (OR) for causal effect on pancreatic cancer, with red indicating increased risk (OR >1) and green indicating protective effects (OR <1). The outer ring shows the statistical significance (*p*-value) of each association, with red segments denoting *p* < 0.05. Taxa with significant causal effects are highlighted in red along the outer edge.

**Figure 4:**
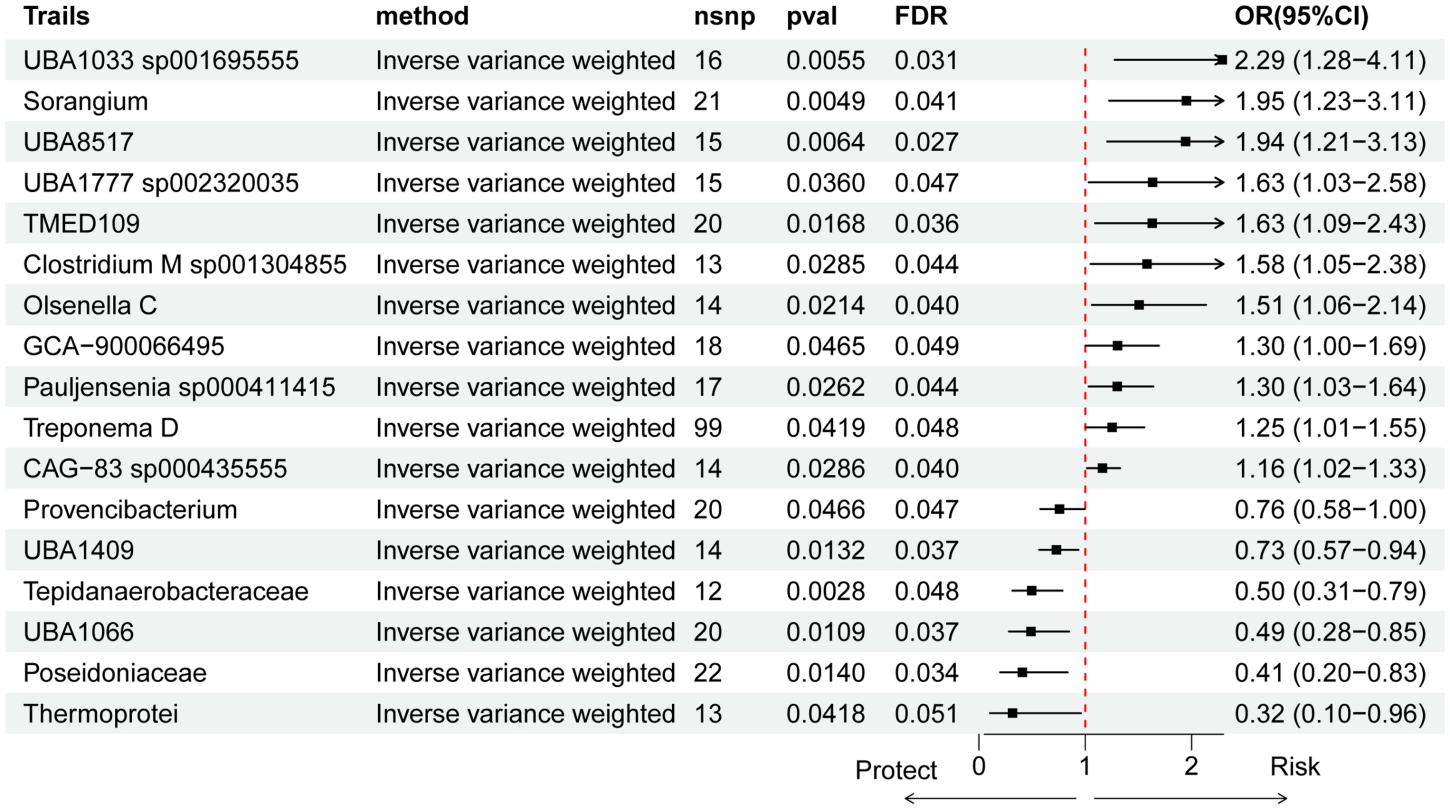
Forest plot of causal effects of gut microbial taxa on pancreatic cancer risk estimated by IVW Mendelian randomization analysis. Each row represents a microbial taxon at genus or higher level. The OR and 95% CI are shown for each association. Horizontal lines indicate the 95% CI, with black squares representing the OR estimate. Dashed vertical line marks OR = 1 (no effect). Taxa with statistically significant associations (*p* < 0.05) are indicated by red dots, and FDR-adjusted *p*-values are reported. Positive ORs indicate increased risk; negative ORs would indicate protective effects.

To further investigate the causal relationships between gut microbiota and pancreatic cancer, we first performed forward MR analysis, which identified 17 bacterial taxa significantly associated with pancreatic cancer risk. A circular heatmap was generated to visually summarize all findings (Figure 3), with the IVW method used as the primary analytical approach (Figure 4). Bacterial taxa are reported using GTDB release 89 nomenclature. Specifically, 11 taxa were associated with an increased risk of pancreatic cancer: CAG-83 sp000435555 (OR = 1.16, 95% CI: 1.02–1.33; *p* = 0.029), Clostridium M sp001304855 (OR = 1.58, 95% CI: 1.05–2.38; *p* = 0.029), GCA-900066495 (OR = 1.30, 95% CI: 1.00–1.69; *p* = 0.047), Olsenella C (OR = 1.51, 95% CI: 1.06–2.14; *p* = 0.021), Pauljensenia sp000411415 (OR = 1.30, 95% CI: 1.03–1.64; *p* = 0.026), TMED109 (OR = 1.63, 95% CI: 1.09–2.43; *p* = 0.017), Treponema D (OR = 1.25, 95% CI: 1.01–1.55; *p* = 0.042), UBA1033 sp001695555 (OR = 2.29, 95% CI: 1.28–4.11; *p* = 0.006), UBA1777 sp002320035 (OR = 1.63, 95% CI: 1.03–2.58; *p* = 0.036), and UBA8517 (OR = 1.94, 95% CI: 1.21–3.13; *p* = 0.006).

Conversely, six gut microbial taxa showed protective associations against pancreatic cancer, of which five remained significant after FDR correction. These included Poseidoniaceae (OR = 0.41, 95% CI: 0.20–0.83; *p* = 0.034), Provencibacterium (OR = 0.76, 95% CI: 0.58–0.99; *p* = 0.047), Tepidanaerobacteraceae (OR = 0.50, 95% CI: 0.31–0.79; *p* = 0.002), UBA1066 (OR = 0.49, 95% CI: 0.28–0.85; *p* = 0.011), and UBA1409 (OR = 0.73, 95% CI: 0.57–0.94; *p* = 0.013). Sensitivity analyses indicated that Treponema D exhibited significant horizontal pleiotropy, as revealed by Cochran’s *Q* test and MR-Egger regression intercept analysis. Despite this, scatter plots and leave-one-out analysis further supported the robustness of the remaining associations (Supplementary Figures S2, S3). Additionally, MR-PRESSO analysis detected no outliers.

To assess reverse causation, we conducted reverse MR analyses with pancreatic cancer as the exposure. Significant reverse causal associations were observed for Treponema D and UBA1409 (*p* < 0.05), indicating potential bidirectional effects. To minimize bias from reverse causality, these two taxa were excluded from further interpretation in the forward MR analysis.

### Causal relationships between serum metabolites and pancreatic cancer

To explore the potential mechanisms underlying the relationship between gut microbiota and pancreatic cancer, we conducted a two-step mediation MR analysis on serum metabolites. Initially, we performed a two-sample MR analysis to investigate the causal relationships between serum metabolites and pancreatic cancer. Subsequently, we assessed the causal impact of gut microbiota on significant serum metabolites.

**Figure 5:**
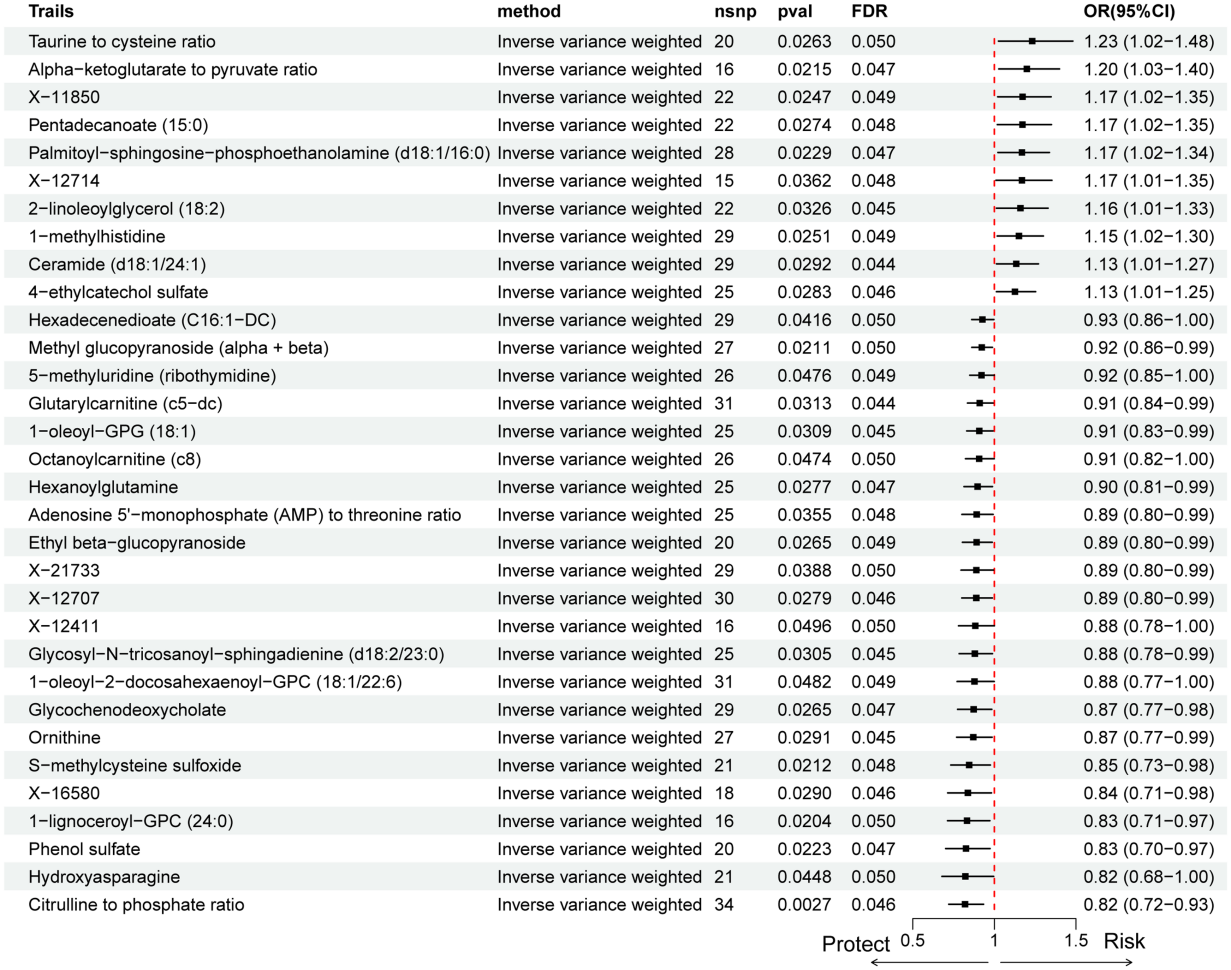
Forest plot of causal effects of serum metabolites on pancreatic cancer risk estimated by IVW Mendelian randomization analysis. Each row represents a metabolite or metabolic ratio. Odds ratios (OR) and 95% confidence intervals (CI) are shown, with red dots indicating statistically significant associations (*p* < 0.05). The dashed vertical line marks OR = 1 (no effect).

MR analysis of 1,400 serum metabolites, with correction for multiple testing using the FDR, identified 10 metabolites as risk factors for pancreatic cancer (Figure 5): taurine to cysteine ratio, alpha-ketoglutarate to pyruvate ratio, X-11850, pentadecanoate (15:0), palmitoyl-sphingosine-phosphoethanolamine (d18:1/16:0), X-12714, 2-linoleoylglycerol (18:2), 1-methylhistidine, ceramide (d18:1/24:1), and 4-ethylcatechol sulfate. Conversely, 22 metabolites were identified as protective factors against pancreatic cancer: hexadecenedioate (C16:1-DC), methyl glucopyranoside (alpha + beta), 5-methyluridine (ribothymidine), glutarylcarnitine (C5-DC), 1-oleoyl-GPG (18:1), octanoylcarnitine (c8), hexanoylglutamine, adenosine 5′-monophosphate (AMP) to threonine ratio, ethyl beta-glucopyranoside, X-21733, X-12707, X-12411, glycosyl-N-tricosanoyl-sphingadienine (d18:2/23:0), 1-oleoyl-2-docosahexaenoyl-GPC (18:1/22:6), glycochenodeoxycholate, ornithine, S-methylcysteine sulfoxide, X-16580, 1-lignoceroyl-GPC (24:0), phenol sulfate, hydroxyasparagine, and citrulline to phosphate ratio.

Sensitivity analyses using Cochran’s *Q* test and MR-Egger regression intercept showed no significant heterogeneity or horizontal pleiotropy. However, reverse MR analysis using the IVW method indicated a significant reverse causal association between phenol sulfate and pancreatic cancer (*p* < 0.05), suggesting potential bidirectional effects. These results highlight a complex metabolic profile associated with pancreatic cancer risk.

### Gut microbiota-mediated effects on pancreatic cancer via serum metabolites

**Figure 6:**
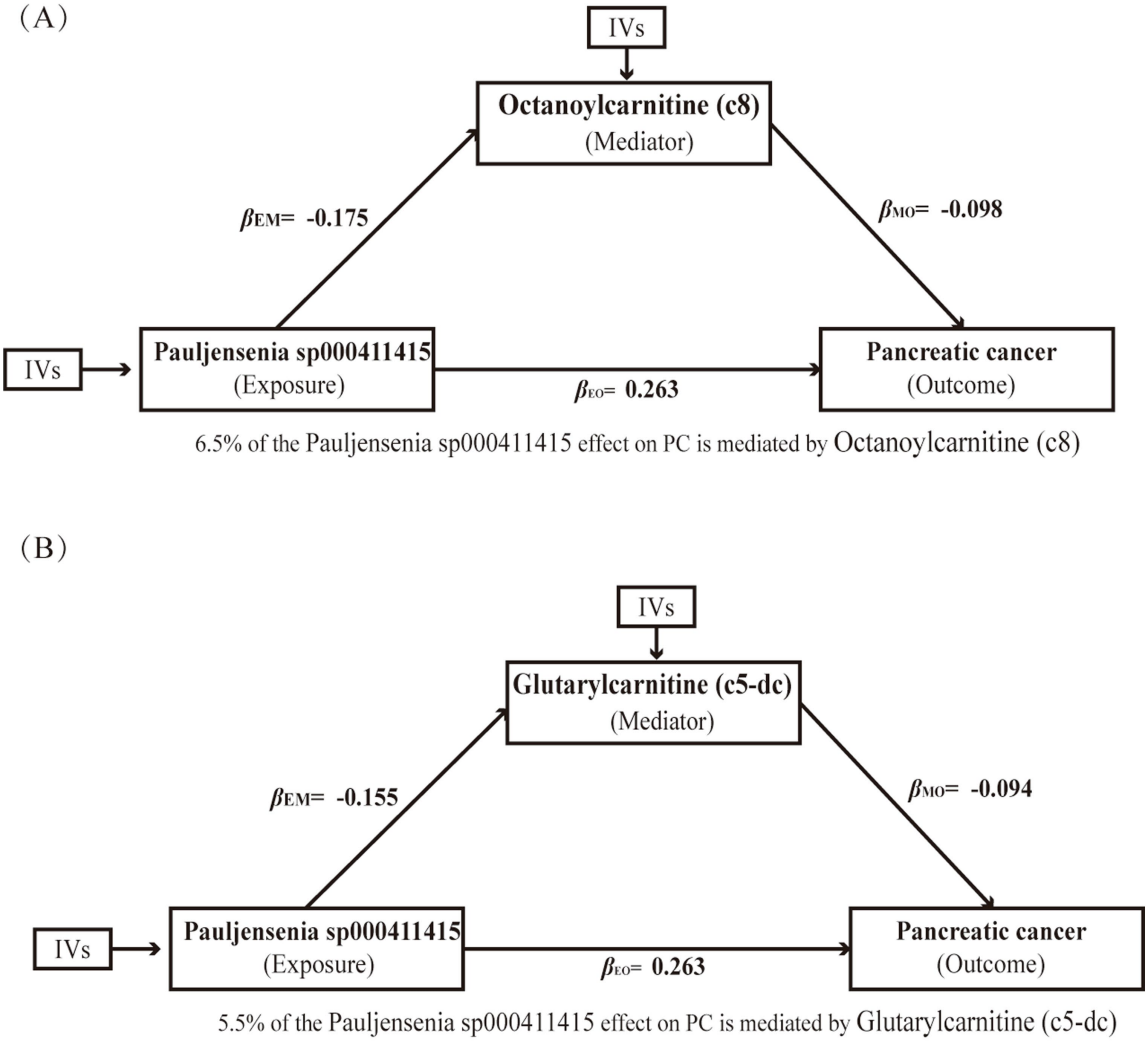
Mediation analysis showing the role of serum metabolites as mediators in the causal pathway from gut microbiota to pancreatic cancer risk. **(A)** Octanoylcarnitine (C8) mediates 6.5% of the effect of *Paulijennia* sp000411415 on pancreatic cancer. **(B)** Glutarylcarnitine (C5-DC) mediates 5.5% of the effect. Solid arrows indicate significant associations (*p* < 0.05); *β* coefficients are shown for each path. *β*_EM_, effects of exposure on mediator; *β*_MO_, effects of mediator on outcome; *β*_EO_, effects of exposure on outcome.

We further identified significant causal relationships between specific gut microbial taxa and serum metabolites. Notably, a decrease in Pauljensenia sp000411415 was associated with elevated levels of octanoylcarnitine (c8) and glutarylcarnitine (C5-DC) (*β* = −0.175 and −0.155, FDR = 0.032 and 0.034, respectively) (Supplementary Table S5). Integrating these findings with the earlier MR results for metabolites and pancreatic cancer, we observed that higher levels of both octanoylcarnitine (c8) and glutarylcarnitine (C5-DC) are causally linked to a reduced risk of pancreatic cancer. To formally test for mediation, we applied the product-of-coefficients method, which confirmed that octanoylcarnitine (c8) and glutarylcarnitine (C5-DC) partially mediate the effect of Pauljensenia sp000411415 on pancreatic cancer risk. Specifically, 6.5% via octanoylcarnitine (c8) (indirect effect = 0.017) and 5.5% via glutarylcarnitine (C5-DC) (indirect effect = 0.015) (Figure 6 and Table 2). Given that acylcarnitines like octanoylcarnitine (c8) and glutarylcarnitine (C5-DC) are critical for mitochondrial fatty acid oxidation and their depletion is linked to metabolic stress and inflammation—a known driver of pancreatic carcinogenesis—our findings provide quantitative evidence that Pauljensenia sp000411415 may promote pancreatic cancer risk, in part, by suppressing systemic acylcarnitine levels.

**Table 2:** The mediation effect of gut microbiota on pancreatic cancer via affecting serum metabolites.

Exposure	Mediation	Outcome	*β* (EM)	*β* (MO)	*β* (EO)	Indirect effect	Direct effect	Direct effect OR (95% CI)
Pauljensenia sp000411416	Octanoylcarnitine (c8)	Pancreatic cancer	−0.175	−0.098	0.263	0.017	0.246	1.0172 (0.9945, 1.0404)
Pauljensenia sp000411417	Glutarylcarnitine (C5-DC)	Pancreatic cancer	−0.155	−0.094	0.263	0.015	0.249	1.0146 (0.9957, 1.0340)

*β* (EM): coefficient from exposure to mediator; *β* (MO): coefficient from mediator to outcome; *β* (EO): coefficient from exposure to outcome (total effect). Indirect effect: product of *β* (EM) and *β* (MO). Direct effect: total effect minus indirect effect; OR (95% CI): odds ratio for direct effect with 95% confidence interval. All estimates were derived using inverse variance-weighted Mendelian randomization.

### Validation of causal associations in an East Asian context using population-matched data

Given the potential limitations of applying European-derived genetic instruments to infer causal effects in a Chinese discovery cohort, we sought to validate our findings using population-matched data. We performed a two-sample Mendelian randomization analysis to assess the causal effects of the differentially abundant gut microbial taxa—identified in our Chinese cross-sectional cohort—on pancreatic cancer risk in East Asian populations.

**Figure 7:**
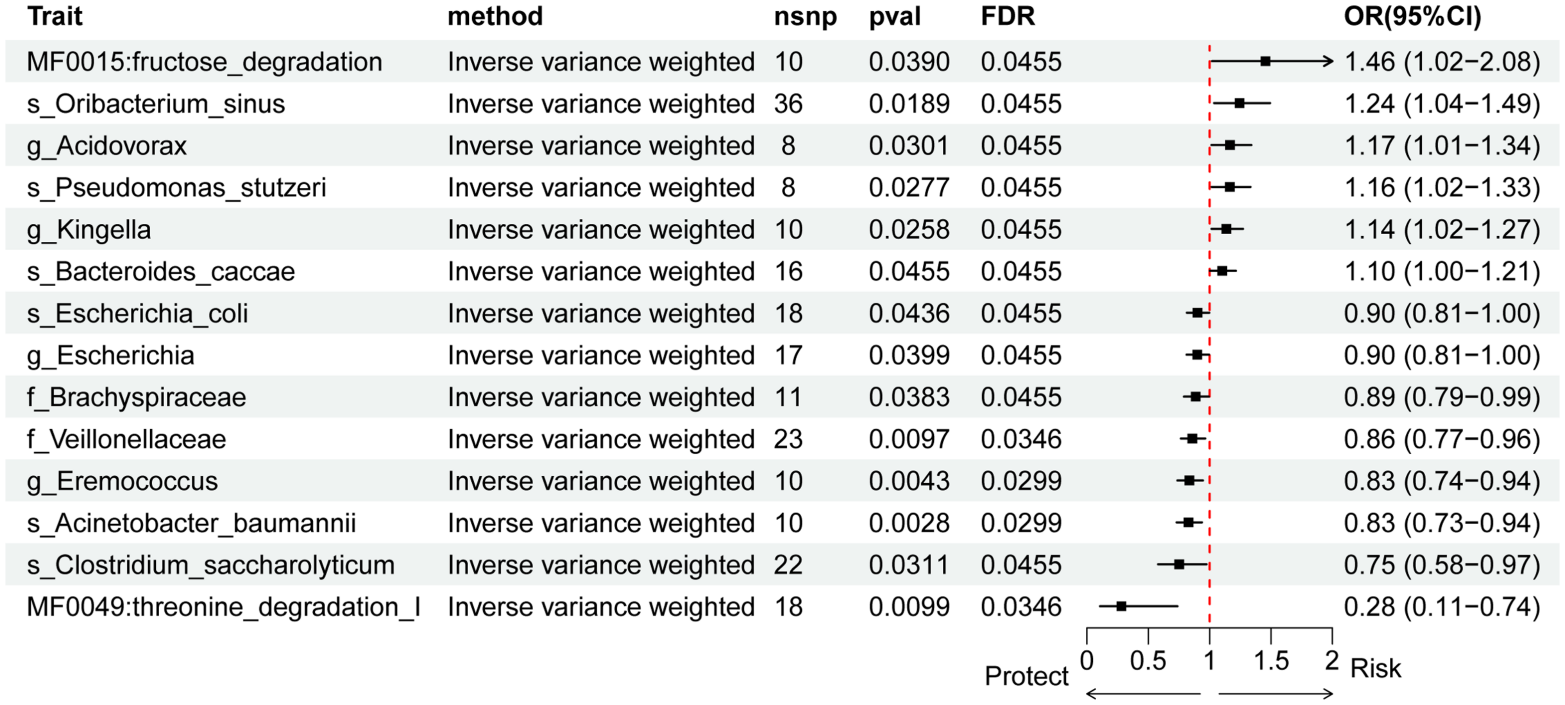
Forest plot of causal effects of gut microbial taxa on pancreatic cancer risk in East Asian populations, estimated by inverse variance-weighted Mendelian randomization analysis. Each row represents a microbial taxon at genus or higher level. Odds ratios (OR) and 95% confidence intervals (CI) are shown for the association between each taxon and pancreatic cancer. Horizontal lines indicate 95% CIs; results are based on genetic instrumental variables derived from Chinese cohort data and East Asian GWAS of pancreatic cancer.

Using genetic instruments derived from East Asian GWAS, we identified 12 microbial taxa and 2 metabolic pathways significantly associated with pancreatic cancer risk (Figure 7). Among them, 8 taxa and one pathway were inversely associated with disease risk and thus considered potential protective factors. These included s_Clostridium_saccharolyticum, s_Acinetobacter_baumannii, g_Eremococcus, f_Veillonellaceae, f_Brachyspiraceae, g_Escherichia, s_Escherichia_coli, and the threonine degradation I pathway. In contrast, 4 microbial taxa and one metabolic pathway were positively associated with pancreatic cancer risk, indicating potential risk factors. These were s_Bacteroides_caccae, g_Kingella, s_Pseudomonas_stutzeri, g_Acidovorax, s_Oribacterium_sinus, and the fructose degradation pathway. Sensitivity analysis using MR-Egger intercept and MR-PRESSO detected evidence of horizontal pleiotropy for s_Oribacterium_sinus (*p* < 0.05), suggesting that the observed association may be influenced by pleiotropic effects.

## Discussion

The human gut harbors one of the most complex and densely populated microbial ecosystems, collectively known as the gut microbiota, which has co-evolved in a symbiotic relationship with its host (Athanasopoulou et al., 2023). Beyond its role in digestion and nutrient metabolism, the gut microbiome functions as a dynamic metabolic and immunomodulatory organ, producing a vast array of bioactive molecules—including neurotransmitters, organic acids, and immune mediators—that can influence host physiology both locally and at distant sites (Kim et al., 2024). Disruption of this finely tuned microbial balance, a state termed dysbiosis, has been increasingly implicated as a potential trigger or amplifier in the development of chronic diseases, including cancer (Asseri et al., 2023). In the context of pancreatic cancer, emerging evidence suggests that alterations in gut microbial composition and function may play a significant role in both tumor initiation and progression (Guo and Shao, 2025).

PC remains one of the most lethal malignancies worldwide, characterized by intricate pathogenesis and a paucity of early-detection biomarkers and curative therapies (Siegel et al., 2021). Here, we integrated microbiome profiling with rigorous causal-inference algorithms and mediation analysis to delineate the contribution of the gut microbiota to pancreatic carcinogenesis. We provide convergent evidence that microbial dysbiosis is not merely an epiphenomenon but functionally implicated in tumor initiation and progression through defined microbial taxa and metabolite-signaling axes, thereby unveiling tractable targets for mechanistic dissection and therapeutic intervention. Our findings not only confirm the presence of profound microbial dysbiosis in patients but also move beyond association to identify specific microbes and metabolite pathways with causal relevance, offering new insights into potential mechanisms and intervention strategies.

The gut microbial ecosystem in PC patients exhibits a consistent pattern of structural and functional dysbiosis, marked by depletion of butyrate-producing Firmicutes—such as Faecalibacterium, Roseburia, and Lachnospira—and expansion of pro-inflammatory genera including Escherichia–Shigella, Veillonella, and Fusobacterium. This pattern is corroborated by a recent European study linking reduced Lachnospira abundance to intraductal papillary mucinous neoplasm (IPMN), a key PC precursor (Sidiropoulos et al., 2024). Strikingly, many of these taxa are also enriched in other microbial niches. Oral microbiome studies report elevated Prevotella, Rothia, Veillonella, Porphyromonas, Actinomyces, and Fusobacterium in PC patients (Meng et al., 2025; Uguz et al., 2025), while tumor tissue analyses have detected increased abundances of *Escherichia coli* and Shigella—members of the Escherichia–Shigella complex identified in our cohort—particularly in pancreatic head cancers (Tajpara et al., 2025). Together, these multi-compartment observations suggest systemic selection of specific bacterial lineages across anatomically distinct sites under shared immunometabolic pressures of malignancy, underscoring a broader paradigm in gastrointestinal carcinogenesis: a systemic shift from a symbiotic to an inflammogenic microbiota (Mori et al., 2018; Pötgens et al., 2018). Butyrate and other short-chain fatty acids (SCFAs) are critical for maintaining intestinal barrier function, regulating T-regulatory cell differentiation, and suppressing NF-κB signaling (Jin et al., 2025; Zheng and Xu, 2025; Xu et al., 2023). Their reduction may facilitate systemic inflammation and immune dysregulation, creating a permissive environment for tumor initiation and progression. The pronounced reduction of Faecalibacterium—a dominant anti-inflammatory commensal typically enriched in healthy individuals—aligns with findings in pancreatic and colorectal cancers (Nagata et al., 2022; Li J. et al., 2024); notably, reduced SNP density in its predominant species *F. prausnitzii* has been observed in specific pancreatic cancer subgroups (Han et al., 2025), hinting at altered strain-level dynamics beyond simple abundance loss.

While 16S rDNA profiling captures microbial community shifts associated with PC, it cannot distinguish whether these changes are drivers or consequences of disease. To address this, we applied two-sample MR to investigate causal relationships between gut microbiota and PC risk, identifying in European populations 17 bacterial taxa with significant causal associations—11 conferring increased risk and 6 exerting protective effects. Notably, among the taxa showing differential abundance in our 16S analysis, Olsenella—which was enriched in PC patients—also demonstrated a significant causal effect on elevated PC risk in MR analysis. This concordance between observational and causal evidence strengthens the plausibility of Olsenella as a potential contributor to pancreatic carcinogenesis. Olsenella, a genus within the Actinobacteriota phylum, has been previously associated with inflammatory conditions such as periodontitis, osteoporosis, and colorectal cancer (Vogtmann et al., 2025; Sheng et al., 2019; Qin et al., 2021; Dewhirst et al., 2001). A key characteristic of this genus is its capacity to produce lactate as a major fermentation product and to degrade mucin (Kraatz et al., 2011; Barandouzi et al., 2020). The overproduction of lactate may acidify the local gut environment, potentially favoring the expansion of other pathobionts and disrupting the symbiotic microbial community structure. More critically, mucin degradation by Olsenella is likely to compromise the integrity of the gastrointestinal mucus barrier—a crucial first line of host defense (Li S. et al., 2024). This barrier impairment could facilitate the translocation of microbial metabolites or even live bacteria into systemic circulation, potentially reaching the pancreas via the portal vein or enterohepatic circulation (Wang et al., 2019). This process may, in turn, trigger chronic systemic inflammation and activate immune cells, thereby fostering a pro-tumorigenic microenvironment that could promote pancreatic carcinogenesis (Mager et al., 2020).

In contrast to abundance-altered, causal taxa like Olsenella, our MR findings unveil a subtler mechanism: microbial influence on PC can operate through functional shifts independent of population size, a paradigm exemplified by Pauljensenia sp000411415. Mediation analysis further revealed that increased abundance of Pauljensenia sp000411415 was positively associated with PC risk, and this effect was partially mediated by its association with reduced levels of circulating octanoylcarnitine (C8) and glutarylcarnitine (C5-DC). This identified Pauljensenia-acylcarnitine axis reveals a novel microbiome-host metabolic interaction mechanism influencing PC risk. While the precise biological functions of Pauljensenia sp000411415 remain incompletely characterized, its genetically predicted link to decreased octanoylcarnitine (C8) and glutarylcarnitine (C5-DC) levels suggests a potential role in disrupting mitochondrial fatty acid β-oxidation and energy metabolism. Medium-chain acylcarnitines, such as octanoylcarnitine (C8), participate in medium-chain fatty acid metabolism, which may originate from dietary sources or microbial fermentation (Solo et al., 2025). Glutarylcarnitine (C5-DC) is associated with lysine and tryptophan degradation pathways (Li Q. et al., 2021). Beyond serving as metabolic intermediates, these specific acylcarnitines may exert direct protective effects—for instance, by supplying alternative energy substrates to damaged tissues, alleviating oxidative stress through enhanced mitochondrial efficiency, or modulating immune cell function and differentiation (Brosnan and Brosnan, 2009; Waagsbø et al., 2016). We propose that Pauljensenia sp000411415 may promote PC development by persistently depleting these functional metabolites, thereby compromising host metabolic homeostasis and tissue repair capacity. These findings highlight a gut microbiota-acylcarnitine-host metabolic axis as a potential pathogenic mechanism in pancreatic carcinogenesis.

However, we also observed substantial population-specific associations. Several microbial taxa showed significant causal effects exclusively in European populations (e.g., Pauljensenia sp000411415, UBA1033 sp001695555) or East Asian populations (e.g., s_Escherichia_coli, s_Acinetobacter_baumannii). Notably, while the specific taxa differed between ancestries, several microbial signals exhibited directionally concordant effects—such as enrichment of pro-inflammatory or mucus-degrading genera and depletion of butyrate producers—suggesting convergent dysbiosis patterns along shared pathogenic pathways. This heterogeneity likely reflects differences in genetic background, dietary patterns, and environmental exposures between populations. For example, the higher intake of plant-based fibers and fermented foods in traditional East Asian diets may promote the colonization or metabolic activity of beneficial microbial groups—particularly butyrate-producing taxa such as Faecalibacterium and Roseburia—which are known to support intestinal barrier integrity and immune homeostasis (Chen H. et al., 2024; Li G. et al., 2021). In our Beijing cohort, these taxa were markedly depleted in pancreatic cancer patients, consistent with a loss of protective functions that may be shaped by long-term dietary habits. Concurrently, population-specific genetic variants in host genes involved in mucosal immunity or bile acid metabolism can shape distinct host–microbiota interaction landscapes, thereby giving rise to ancestry-dependent causal associations (Graves and Milovanova, 2019). Importantly, the absence of exact taxonomic overlap does not undermine causality; rather, it underscores the necessity of accounting for population-specific factors in microbiota-targeted strategies for cancer prevention and treatment. Precision medicine initiatives should incorporate inter-population differences in gut microbiota composition and host–microbe interactions to develop tailored interventions that maximize therapeutic efficacy while minimizing unintended risks.

It is also instructive to contrast our MR findings with the 16S results. For instance, while MR suggested protective effects of f_Veillonellaceae and g_Escherichia (s_Escherichia_coli), their unclassified counterparts—g__Veillonellaceae_unclassified and g__Escherichia-Shigella—were enriched in pancreatic cancer patients. Conversely, s_Bacteroides_caccae was identified as a risk factor in MR, yet other Bacteroides species showed reduced abundance in disease. These inconsistencies likely reflect the distinction between causal drivers and disease-associated ecological shifts. MR captures lifelong genetic predisposition to microbial abundance, reflecting upstream influences on carcinogenesis. In contrast, cross-sectional 16S data may capture downstream consequences of pancreatic dysfunction—such as biliary obstruction or immune suppression—that favor opportunistic colonization regardless of initial causality. Moreover, intra-genus functional heterogeneity means that not all members of a taxon share the same biological role. Thus, microbial enrichment in disease does not imply pathogenicity, reinforcing the need for causal inference to disentangle true contributors from bystanders.

Notably, our 16S rDNA gene-based approach is inherently limited to bacterial profiling and cannot assess changes in the virome or mycobiome. Nevertheless, fungi and viruses—through cross-kingdom interactions—are increasingly recognized as key modulators of bacterial community structure and host immunity, potentially influencing pancreatic carcinogenesis (Meng et al., 2025). Our team is currently conducting large-scale shotgun metagenomic sequencing studies to enable a more comprehensive, multi-kingdom characterization of the gut microbiome in pancreatic cancer.

Several limitations should be acknowledged. First, although we employed multiple sensitivity methods (e.g., MR-Egger, weighted median, MR-PRESSO) to mitigate horizontal pleiotropy, residual confounding through alternative pathways cannot be fully excluded. Second, our two-sample MR relies on summary-level GWAS data, which precludes adjustment for individual covariates, assessment of non-linear effects, and direct inference of temporality—highlighting the need for longitudinal validation (Visscher et al., 2017). Third, while we leveraged both European and East Asian datasets, population-specific genetic and environmental factors may limit generalizability to other ancestries. Fourth, our observational 16S cohort is modest in size and enriched for late-stage disease (only 6 early-stage cases), limiting power to detect stage-related microbial differences; findings require replication in larger, stage-stratified cohorts. Finally, although mediation analysis implicates a Pauljensenia–acylcarnitine axis in pancreatic cancer risk, its causal mechanism awaits functional validation in experimental models.

In conclusion, our integrative analysis reveals that gut microbiota influences pancreatic cancer risk through both compositional shifts and causal microbial effects, exemplified by the dual-evidence role of Olsenella, the functionally mediated protection of Pauljensenia, and the complex interplay between causal effects and disease-associated dysbiosis. These findings advance our understanding of the gut–pancreas axis and highlight the potential of combining observational and causal inference approaches to identify novel targets for early intervention and microbiome-based therapeutics.

## Data Availability

The data presented in this study are publicly available. The data can be found here: https://ngdc.cncb.ac.cn/gsa/browse/CRA031736. GSA Accession Number: CRA031736.

## Footnotes

1
https://github.com/MRCIEU/TwoSampleMR
