# Supplementary material for: Gut microbiota, circulating metabolites, and pancreatic cancer risk: a multi-method causal inference study with cross-population validation
Source: Front Microbiol. 2026 Jan 23;16:1730313. doi: 10.3389/fmicb.2025.1730313 (PMC12876191; doi:10.3389/fmicb.2025.1730313)
Supplement: Supplementary file 1 [file Supplementary_file_1.docx]

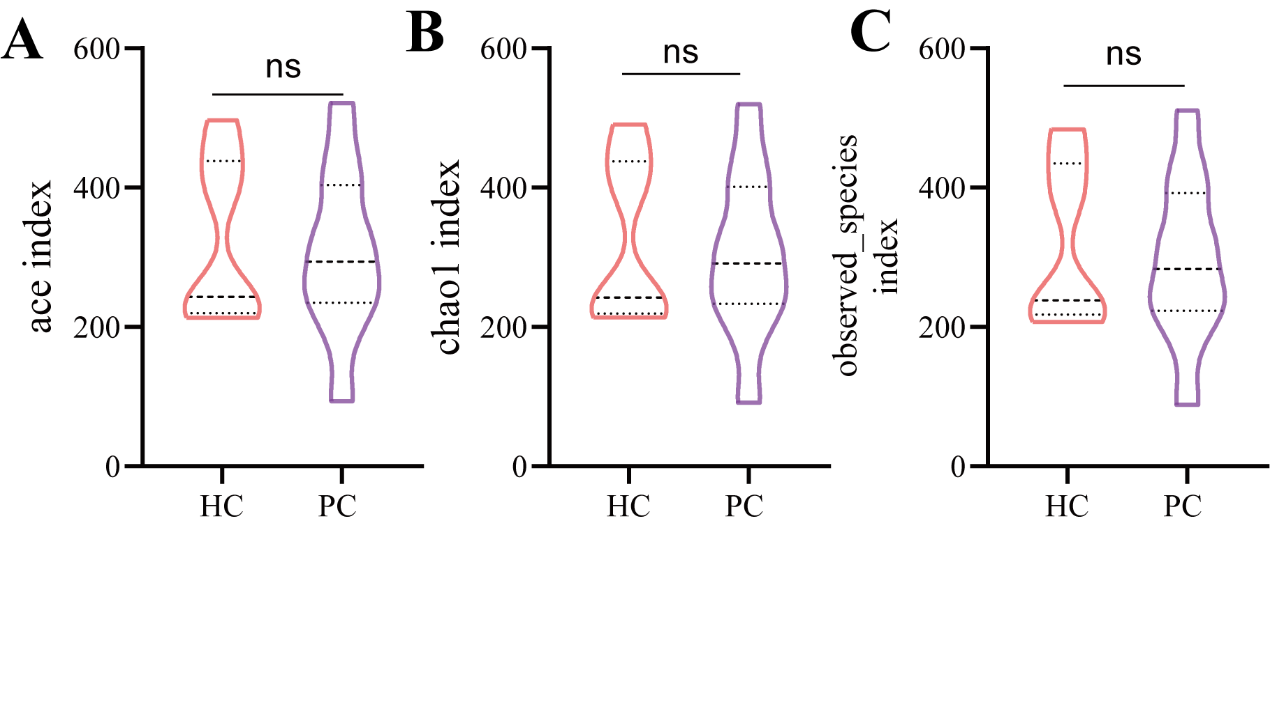


**S1. Comparison of gut microbial alpha diversity between healthy controls (HC) and pancreatic cancer patients (PC).**

(A–C) Violin plots showing the distribution of three alpha-diversity indices: ACE index (A), Chao1 index (B), and observed species index (C). No statistically significant differences were observed between HC and PC groups for any index (*P* > 0.05, Mann–Whitney U test; ns = not significant).


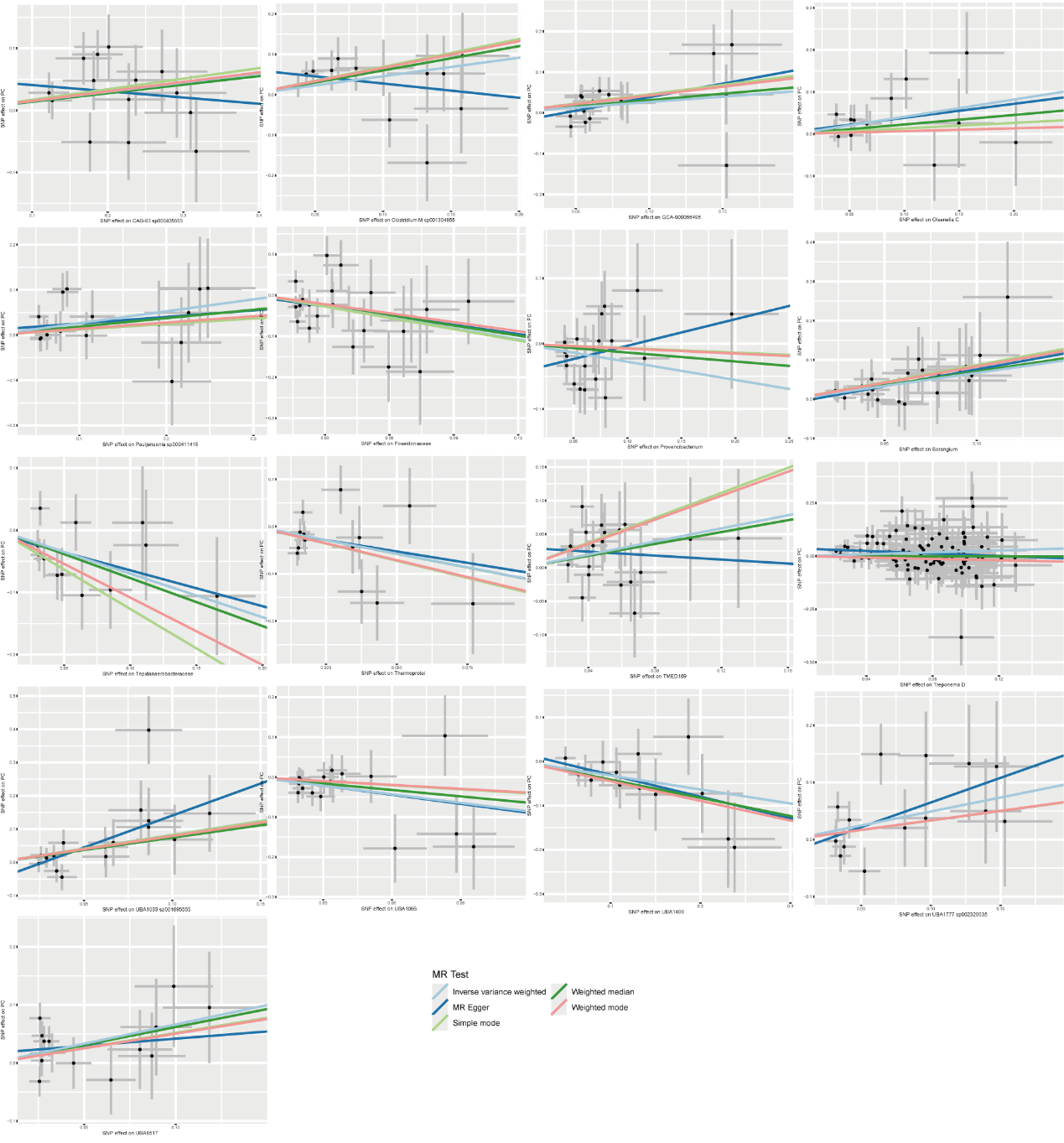


**S2.Scatter plot of Mendelian randomization estimates for gut microbiota–pancreatic cancer association.**

Each point represents a single SNP associated with the microbial taxon of interest, plotted by its effect size on the exposure (x-axis) and on the outcome (y-axis). The direction and magnitude of the causal estimate are indicated by the slope of the regression line (solid black line), with confidence intervals shown as shaded region. The horizontal dashed line indicates the null effect (β = 0).


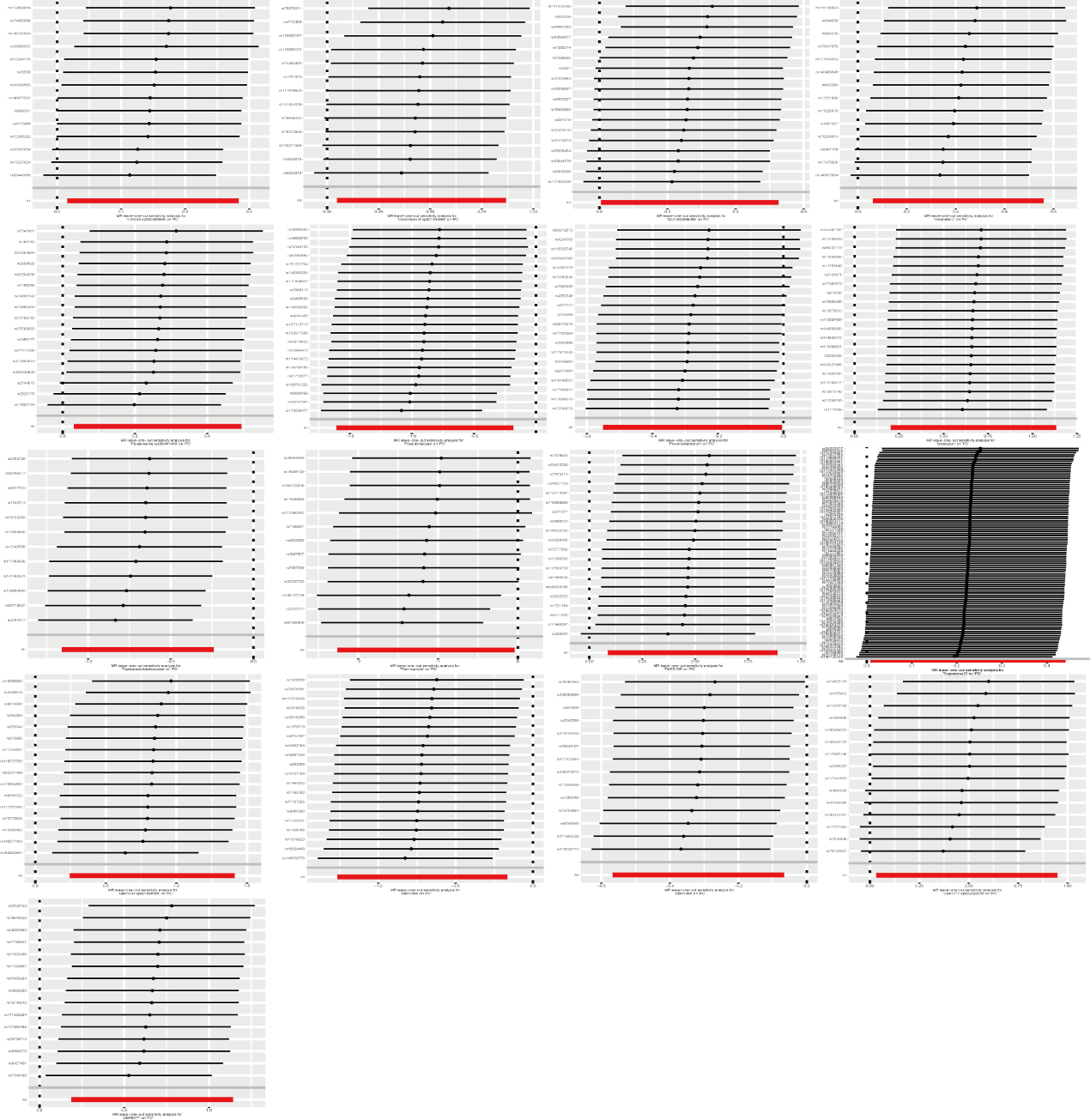


**S3 Leave-one-out sensitivity analysis.**

This panel shows the estimated causal effect of the microbial taxon on pancreatic cancer risk after sequentially removing one SNP at a time. Each dot represents the causal estimate when one SNP is excluded; the central red dot corresponds to the full model estimate.
